# Non-Bovine Milk: Sources and Future Prospects

**DOI:** 10.3390/foods11131967

**Published:** 2022-07-02

**Authors:** Alaa El-Din A. Bekhit, Isam A. Mohamed Ahmed, Fahad Y. Al-Juhaimi

**Affiliations:** 1 Department of Food Sciences, University of Otago, P.O. Box 56, Dunedin 9054, New Zealand; 2 Department of Food Science and Nutrition, College of Food and Agricultural Sciences, King Saud University, P.O. Box 2460, Riyadh 11451, Saudi Arabia; iali@ksu.edu.sa (I.A.M.A.); faljuhaimi@ksu.edu.sa (F.Y.A.-J.)

## 1. Introduction

Milk is the first food that mammals are exposed to. Milk supplies the nutrients required for growth and all the compounds required for the physiological functions of newborns. Apart from the well-known nutritional roles of milk, it provides a wide range of biological compounds that are involved in the modulation of the immune system, bone health, and gut microbiome that collectively are necessary for health and wellbeing. The consumption of non-bovine milk is an old tradition that goes back thousands of years, with the consumption of goat and sheep milk in the Mediterranean region and Arabian Peninsula, as well as in several European and Asian countries; camel milk in the Arabian Peninsula and Mongolia; mare milk in Northern China, Mongolia, and Kazakhstan; and deer milk in the north of Europe and Russia being common practice. With the expansion of cow farming and cows becoming the main source of milk, the use of milk from other species has faded into specialized traditional dairy products. However, lore/traditional wisdom and rapid communications have highlighted the potential health benefits of these non-bovine milk sources as well as the superior quality of certain dairy products produced from them.

Recent changes in food production whereby plant-based foods have been introduced as alternatives to animal-based food products are fueled by interest in healthier food products, more sustainable food production systems, and the need for new foods to meet future increased demands. Milk was among the earliest foods to be produced as an “alternative” product for cultural or health reasons. For example, soy milk has a long history in China and many Asian countries as a food item and has been increasingly used as a popular milk-like due to its lack of lactose and other allergenicity issues associated with cow milk. The menu of plant-based milk-like products now includes products made from sesame, oat, rice, almond, hemp, hazelnut, peanut, cashew, and coconut. More recently, an increasing interest in “Entomilk”, a milk that is produced from edible insects, led to a start-up company (https://gourmetgrubb.com/entomilk/, accessed on 24 June 2022) producing Entomilk ice cream at a commercial scale, with some reports of positive feedback on its products.

## 2. Non-Bovine Milk from Animal Sources

Global milk production worldwide in 2020 (886.9 million tonnes) was 11% higher than that reported in 2016 (798,476,318 tonnes) and more than two and half times that produced in 1961 (344,184,775 tonnes). Buffalo milk production made up 5.2% of the global milk production in 1961, and its production increased to 13.9% and 15.2% in 2016 and 2020, respectively (https://www.fao.org/faostat/en/#search/milk, accessed on 25 June 2022). The percentage of the combined total production of cow and buffalo milk has remained more or less constant at 96% (cow and buffalo milk made up 82.6%, and 13.9% of the world milk production, respectively, in 2016 and 81% and 15.2%, respectively, in 2020). In 2020, goat, sheep, and camel milk represented 2.3%, 1.2%, and 0.4% of the world production (https://www.fao.org/faostat/en/#search/milk, accessed on 25 June 2022). These latter species together made up less than 5% of global milk production but play an important role in the rural economy of several regions in the Mediterranean and Southeast Asia, where they are mostly used in processed dairy products [1]. Deer, donkey, and mare milk are not widely consumed or traded, but they have significant cultural importance in certain communities [2]. Recently, non-bovine milk has been the focus of extensive research activities due to its potential health benefits, as summarized in recent reviews [3,4,5,6], and due to interest in incorporating these types of milk in infant formula [7,8] because of their low allergenicity compared to cow milk.

Milk composition is affected by many factors that are related to animals (species, age, parity, lactation stage, breed, and overall genetic background and health), farming practices (management system, diet, and the number of offspring), and environmental (climate, soil, and altitude) factors. The composition and physicochemical properties of milk from different species have evolved to meet the requirements of their offspring (nutrients, calory input, and digestibility, etc.). The use of milk for human consumption brings into consideration the physicochemical characteristics of the milk that will dictate the functional properties and several subsequent technological (utility, yield, structure, and stability) and sensory properties as well as the value of the milk and its products. Sheep milk is used in several kinds of specialty cheeses among which is Spanish Manchego. Information regarding the impact of sheep breeds on the product quality of dairy products is scarce. The production of Mexican Manchego-style and sensory properties was investigated using hair sheep (e.g., Pelibuey) of Mexican Manchego-style cheese during 6 months of ripening [9]. Extended ripening for 180 days reduced scores for appearance, color, odor, taste, texture, and overall acceptance compared to fresh samples examined at 1 day of ripening or after 90 days of ripening.

### 2.1. Physicochemical Changes

Ruminant milk proteins have been the subject of extensive research, with most of the research focusing on the identification and characterization of cow milk protein due to its wide commercial use and importance. Caseins are the most important proteins in milk contributing to about 80% of the protein of cow milk. Whey proteins, which contribute about 20% of the protein of cow milk, consist mostly of lactoglobulin, lactalbumin, lactoferrin, serum albumin, and immunoglobulins. In the present Special Issue, new information is reported on the effect of milk composition and feed intake, and satiety in a rat model [10]. Changing the milk whey: casein protein ratio from 20:80 to 40:60 affected food intake, glucose metabolism, hormone release, and genes involved in satiety.

The utilization of proteomic tools resulted in the identification of hundreds of proteins. The same level of knowledge on the protein composition of milk from other species is not yet established, except for sheep milk [11]. The protein profile of defatted whole milk of cow, sheep, goat, and red deer milk demonstrated differences in the separation of caseins, lactoglobulin, and lactalbumin [12] and suggested some differences in the hydrophobic interaction characteristics and in amino acid composition.

The high solid contents of sheep, buffalo, and deer milk, especially the protein content, result in different physicochemical properties compared with cow milk (e.g., highest viscosity and micelle mineralization) and can result in a higher cheese yield and higher nutrient density. In particular, interesting properties such as hypo-allergenicity and immunomodulatory activities [3,13,14] have been a catalyst for ongoing research activities.

### 2.2. Health Benefits

Non-bovine milk from animal sources contains substantial quantities of several nutrients such as oligosaccharides, lipids, bioactive peptides, high-quality protein, minerals, and vitamins with nutritional and health benefits. The types, concentration, and composition of these nutrients vary due to breed, animal age and season, feed type and quantity, and environmental conditions [15]. Camel milk fats contain small size fat globules, high levels of lactadherin-like protein, essential fatty acids, unsaturated fatty acids, phospholipids, low amounts of cholesterol, and saturated fatty acids [16]. Camel milk has a high digestibility; good nutritional value; and health effects such as anti-bacterial, anti-inflammatory, and anti-hyperlipidemia activities [16].

The consumption of sheep milk by rats fed a diet restricted in calcium and phosphorous contents was found to increase the levels of calcium, phosphorous, and strontium in the bones of the rats, and thereby it has a good prospect of maintaining bone health in diets low calcium and phosphorous [17].

Human milk oligosaccharides (HMOs) are essential components in human breast milk that are involved in the immune system and brain development and the modification of gut microbiota, and possess antimicrobial effects against pathogens in newborn babies [18]. Sialylated milk oligosaccharides (SMO) are the most important milk oligosaccharides found in human milk and milk of other animals such as bovine, caprine, porcine, elephantine, equine, and donkey which are functionally different among milk of these animals [18]. Among milk from these animals, elephant and goat milk contain high quantities of soluble SMOs and could thus potentially be used in the development of infant formulas [18].

## 3. Non-Bovine Milk from Plant Sources

Plant milk-like products have been enjoying impressive growth recently, with a global sales value of US$ 20 billion in 2021 (https://www.euromonitor.com/article/trends-to-watch-in-plant-based-milk, accessed on 24 June 2022) and with milk contributing more than 90% of the sales. Generally speaking, four different plant groups have been traditionally used or are emerging as sources of milk-like products. Nuts (almond, cashew, hazelnut macadamia, and peanut), seeds (sesame, sunflower, and hemp), legumes (soybean, adzuki, black eye bean, chickpea, and white lima), and grains (adlay, barley, buckwheat, oat rice, quinoa, and millet) are among the popular sources of plant milk-like products. These different sources result in products with different physicochemical nutritional and sensory properties [19]. Soy milk-like has the largest market share (https://www.euromonitor.com/article/trends-to-watch-in-plant-based-milk, accessed on 24 June 2022), but almond and oat milk-like products have been steadily growing in the last 4 years, especially in North America. The main drivers for the growing interest in plant milk-like products are mostly the same associated with plant-based foods (health, environmental and moral motivations). In addition to the sensory attributes of plant milk-like products, interesting cultural, social, and political aspects are associated with these products [20].

## 4. Entomilk

Interest in insect milk was probably triggered by early reports that examined the composition of milk produced by cockroaches (*Diploptera punctata*) [21] that reported its nutritional rich composition (45% protein, 25% carbohydrate, and 16 to 22% lipid). Subsequent media hype considering it as a superfood (https://www.npr.org/sections/thesalt/2016/08/06/488861223/cockroach-milk-yes-you-read-that-right, accessed on 23 June 2022) led to a commercial interest in insect milk. The difficulties faced in obtaining commercial milk products from cockroaches led to the consideration of other edible insects, such as Black Solider larvae (https://gourmetgrubb.com/entomilk/, accessed on 23 June 2022) as well as mealworms [22]. The excitement and novelty of Entomilk appear to be resonating with young consumers and it has the potential to meet sustainable and ethical food production goals, which can only support a positive outlook.

## 5. Conclusions

The future of milk and dairy products appears to be facing significant changes in Western countries with the rise in the consumption of alternative milk products due to health and environmental factors. The consumption of plant-based milk-like in many regions (e.g., the Middle East) is challenged by the unfamiliar sensory attributes of these products and new formulations will be required to improve these aspects. Additionally, the perception of plant-based milk-like products as less nutritious compared to animal milk is a current issue, but this is very likely to change as more information on plant-based milk-like products (digestibility, nutritional properties, health effects…, etc.) becomes available. There is little information known on the nutritional consequences of the long-term consumption of these plant-based products. Of interest is the presence of specialized proteins in animal milk, as these play an important role in binding minerals and bone health. The presence of counterpart molecular systems in plant-based milk-like products is yet to be demonstrated. The future of Entomilk may be difficult to predict in the absence of sufficient information on its composition, digestibility, stability, and the presence of anti-nutrient compounds that may influence its use. Religious beliefs of certain faiths (such as Judaism and Islam) and the disgust/fear of insects can have a major impact on Entomilk, especially in the Middle East.
